# Single‐Step Electrochemical Aptasensor for Melatonin Detection in Unprocessed Plasma

**DOI:** 10.1002/advs.76882

**Published:** 2026-11-04

**Authors:** Hamed Shieh, Arash Khorrami Jahromi, Fernando Gonzales‐Aste, Sajad Shiekh, Roozbeh Siavash Moakhar, Sahar Sadat Mahshid, Sara Mahshid

**Affiliations:** ^1^ Department of Bioengineering McGill University Montreal Quebec Canada; ^2^ Department of Psychiatry Faculty of Medicine McGill University Montreal Quebec Canada; ^3^ Department of Physics McGill University Montreal Quebec Canada; ^4^ Beeta Biomed Clinical Innovation Platform Montreal General Hospital Montreal Quebec Canada; ^5^ Division of Experimental Medicine McGill University Montreal Quebec Canada

**Keywords:** aptamer, circadian rhythm, electrochemical biosensor, melatonin, sleep disorders

## Abstract

Melatonin is a clinically relevant biomarker for diagnosing circadian rhythm sleep disorders (CRSDs) and monitoring circadian disruption associated with cancer, metabolic and cardiovascular disease, and psychiatric disorders. This work reports the implementation of an electrochemical aptamer‐based biosensor (E‐aptasensor) for direct reagentless detection of melatonin within physiological levels. The E‐aptasensor employs an optimized surface population of the aptamers in a conformational change‐based assay to detect melatonin over a broad dynamic range, including the picomolar physiological range. The behavior of the aptameric assay was validated both in bulk solution and in silico using fluorescence resonance energy transfer (FRET) and molecular docking (MD). The E‐aptasensor achieves sensitive and selective melatonin detection, with a limit of detection (LOD) of 0.75 pM and strong specificity against a panel of relevant interferents. Fitting the electrochemical responses to the Hill–Langmuir (HL) binding model yielded an apparent electrochemical dissociation constant (K_d, app_) of 37 pM. Notably, the E‐aptasensor showed promising performance in unextracted human plasma, avoiding the extraction step typically required for immunoassays. These features support future development of rapid melatonin profiling for CRSD diagnosis and monitoring, as well as future point‐of‐care implementation. The single‐step E‐aptasensor offers a promising, faster, and simpler complement to gold‐standard techniques.

## Graphical abstract

Melatonin is a well‐known biomarker with significant potential for clinical use in diagnosing circadian rhythm sleep disorders (CRSDs) and monitoring patients with documented circadian clock disruption. An electrochemical aptamer‐based biosensor (E‐aptasensor), employing an optimized population of melatonin aptamers, has been proposed as a solution for the one‐step, reagentless detection of melatonin within physiological levels.

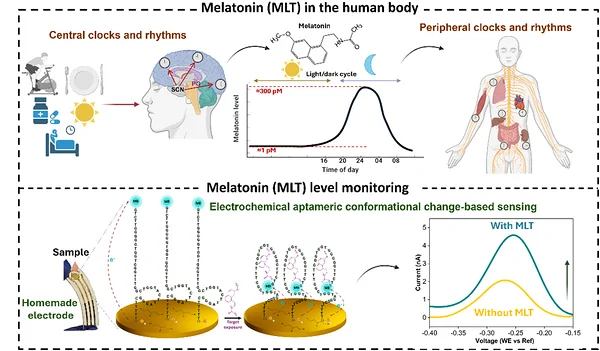

## 1 Introduction

Many human physiological, behavioral, and hormonal functions exhibit circadian rhythms, which are endogenous cycles of periods of about (circa‐) a 24‐h day (‐dian). The integrity of these rhythms has been demonstrated to have a proportional effect on human health. Conversely, the desynchrony of such endogenous cycles with the external environment, also known as circadian misalignment, has been directly linked to multiple health conditions. Some of the most relevant include sleep‐wake disorders, metabolic problems, cardiovascular disease, and cancer [1, 2, 3, 4]. Melatonin (N‐acetyl‐5‐methoxytryptamine, MLT) is an indole hormone produced by the pineal gland (PG) in the brain. Its secretion is under the control of the circadian system, and in humans, melatonin's circulating levels are lowest during the day, at < 2 pg/mL (8 pM), and highest at 30–80 pg/mL (130–350 pM) during the night [5]. It is indeed the timing of the daily rise in melatonin levels under dim‐light conditions, known as dim‐light melatonin onset (DLMO), that has been considered the gold‐standard method for internal circadian phase assessment [6, 7]. DLMO is increasingly being used to monitor circadian misalignment and other CRSDs, such as Delayed Sleep Phase Disorder (DSPD), Advanced Sleep Phase Disorder (ASPD), non‐24‐h sleep‐wake disorder, jet‐lag disorder, and shift‐work disorder [8]. In addition to supporting the diagnosis, monitoring, and treatment of such disorders, the use of DLMO as a reliable circadian phase marker has the potential to open a wide range of personalized therapeutic possibilities, assisting in appropriately timing lifestyle interventions and therapies for many other medical disorders, expanding the field of circadian medicine [9, 10, 11].

Sensitive melatonin measurement has thus been recognized as a crucial need for human well‐being. However, current methods for measuring melatonin, particularly those used to determine DLMO, are logistically demanding, limiting widespread clinical adoption [6]. While DLMO is considered the gold standard for assessing circadian phase in humans, it relies on serial melatonin sampling over multiple hours, often repeated only at weekly or longer intervals, making it impractical for routine clinical use. This challenge was acknowledged in a joint 2021 report from the Sleep Research Society and the Society for Research on Biological Rhythms [8]. Moreover, melatonin is challenging to quantify in biological fluids due to its low concentrations and the presence of potentially interfering compounds [12]. In recent years, a range of analytical assays have been used to detect melatonin, including high‐performance liquid chromatography (HPLC), mass spectrometry, capillary electrophoresis, and gas chromatography, which offer high sensitivity and accuracy but are often too expensive, time‐consuming, and infrastructure‐intensive for routine or point‐of‐care use. Immunoassays such as radioimmunoassay (RIA) and enzyme‐linked immunosorbent assay (ELISA) are therefore widely used for melatonin quantification owing to their established performance and commercial availability [5, 12, 13, 14]. However, without appropriate sample extraction, antibody cross‐reactivity with structurally related analytes and matrix components compromises sensitivity, specificity, and overall accuracy. ELISA, in particular, involves multiple sample‐preparation and incubation steps and has been reported to overestimate melatonin relative to the most sensitive analytical methods [15]. Other methods like RIA require a radioactively labelled antibody that demands specialized handling and disposal [12]. Further, for accurate quantification in complex biofluids (e.g., plasma), extraction using commercial kits is often required to remove metabolites, lipids, and other interferents that can bias antibody‐based readouts and lead to overestimation of melatonin levels [16, 17]. Moreover, generating highly selective antibodies via immunization against low‐molecular‐weight analytes is intrinsically challenging [13, 18]. More sensitive reference techniques, such as HPLC [19] and liquid chromatography‐tandem mass spectroscopy (LC‐MS/MS) [20, 21], provide improved accuracy but typically require sophisticated instrumentation, complex workflows, and trained personnel, and are not cost‐effective for high‐throughput testing [22]. Therefore, these limitations underscore an unmet need for accurate, sensitive, rapid, and practical melatonin biosensors suitable for point‐of‐care settings.

Melatonin biosensors reported in the literature often lack one or more critical features, including a physiologically relevant limit of quantification (LOQ), adequate sensitivity and selectivity, portability, and the ability to perform a simple, single‐step assay [23, 24, 25, 26, 27, 28, 29]. Among various bioassays, aptamer‐based schemes have shown strong potential for measuring small‐molecule biomarkers [30, 31, 32]. Recently, for example, a solution‐phase colorimetric aptamer assay was reported for melatonin quantification [15]. On the other hand, electrochemical transduction, alone or integrated with enzymatic and affinity‐based recognition, has been widely applied to detect diverse small‐molecule targets, yet remains comparatively underexplored and insufficiently developed for melatonin sensing [16, 23, 29, 33, 34, 35, 36]. Redox‐reporter‐modified aptamers tethered to the surface of an electrode are the core of the established electrochemical aptamer‐based biosensor (E‐AB) framework. These reagentless readouts provide signal through alterations in interfacial electron‐transfer behavior upon aptamer‐target interactions [37, 38, 39]. A brief comparison of conventional immunoassays and recently reported melatonin‐sensing platforms has been provided in Table S1. Here, we present an aptamer‐based electrochemical biosensor tailored for melatonin sensing by integrating a melatonin‐binding aptamer as a selective biorecognition element with a custom‐fabricated gold working electrode (GWE) as a sensitive and reproducible transducer. Aptamer‐target‐specific electrochemical/optical responses were also supported by sequence‐specific controls. Importantly, tuning the surface population of aptamers immobilized on the GWE enabled the E‐aptasensor to quantify melatonin across distinct dynamic ranges (spanning 1 pM to 1 mM by modulating surface loading), including the narrow physiological picomolar window (∼1–400 pM). In the present work, cost‐effective homemade GWE platforms were mass‐produced using stencil lithography and additive manufacturing, eliminating the need for complex microfabrication workflows or expensive commercial electrodes. The GWE format is compatible with both commercial and homemade potentiostats [40], supporting its potential for future point‐of‐care applications. The E‐aptasensor was developed based on an aptameric conformational change‐based assay. In this approach, methylene blue (MB)‐tagged aptamers immobilized on the GWE undergo conformational changes upon binding to melatonin molecules. These structural changes alter the distance/apparent electron‐transfer behavior between MB (used as the redox probe) and the GWE surface, resulting in measurable current changes detected by the electrochemical transducer. The resulting peak current variations, recorded by the rapid and straightforward square wave voltammetry (SWV) technique, correlate directly with the concentration of melatonin present. Additionally, the conformational changes of the melatonin aptamers upon melatonin binding were qualitatively supported using FRET and in silico MD analysis. While FRET provided experimental evidence of distance changes in solution, the docking simulations offered preliminary structural insights; however, these simulations are qualitative and do not fully capture the dynamics of surface‑immobilized aptamers in the electrochemical biosensor configuration. Finally, the optimized melatonin E‐aptasensor was validated in real human plasma pre‐characterized by RIA, and its selectivity was evaluated against relevant potential interferents. Accordingly, the present study leverages the established E‐AB architecture and tunes it for physiological‐range melatonin detection in untreated plasma, using a low‐cost electrode platform that is compatible with readily available portable measurement instruments [41, 42]. While the primary focus of this study is the analytical development and optimization of the E‐aptasensor, its application to time‐resolved sampling and measurement protocols, such as DLMO analysis, remains the subject of future work. In the present study, “one‐step” refers to the direct exposure of a pre‐functionalized electrode to the target solution, followed by electrochemical readout, without any dedicated sample‐preparation step or the addition of reagents.

Figure 1 schematically shows the human circadian system. It also demonstrates the comparison between lengthy and complex gold‐standard measurement methods of melatonin and the proposed E‐aptasensor. The novel combination of the MB‐tagged melatonin aptamer, used in a conformational change‐based assay format, and the homemade GWE platform provided reliable and outstanding performance and showed potential for its integration into a POC system.

**FIGURE 1:**
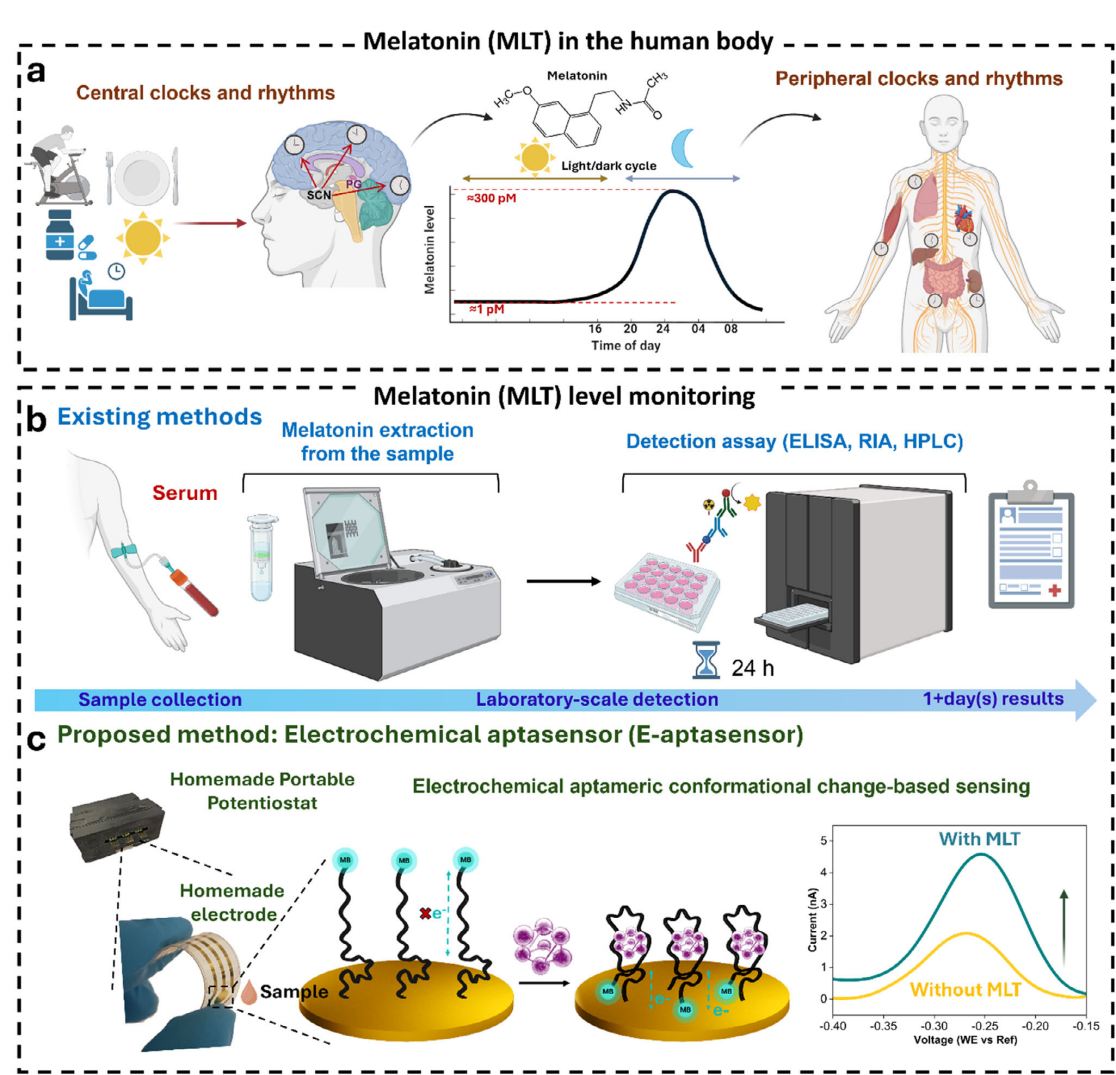
Schematic of the human circadian system and comparison between the gold‐standard techniques and the E‐aptasensor for melatonin detection. (a) Environmental and behavioral cues (light, activity, meals, sleep) entrain the suprachiasmatic nucleus (SCN), which drives pineal gland (PG) melatonin secretion and synchronizes peripheral clocks across organs; when these rhythms lose coordination, sleep–wake cycles and many other physiological functions are adversely affected. Consequently, melatonin acts as a key humoral signal that regulates and synchronizes peripheral biological functions. (b) Existing protocol for melatonin level monitoring includes complex procedures including (i) sample collection and preparation, (ii) laboratory‐based equipment, and (iii) hours of operation and reporting. (c) Proposed electrochemical workflow for melatonin sensing, including (i) inexpensive flexible reproducible GWEs providing a robust platform for aptamer immobilization (these electrodes are compatible with portable potentiostats including a modified version of the homemade device previously developed by our group [40]), (ii) the direct, reagentless aptameric assay undergoing conformational changes upon binding to melatonin, (iii) providing the signal change in the electrochemical measurement.

## 2 Results and Discussion

This study focuses on the implementation and optimization of an E‐aptasensor for melatonin detection, with particular emphasis on investigating the target‐binding (capture) mechanism of the candidate aptamers. Therefore, before integrating the melatonin aptameric assay into the biosensing platform, its proposed mechanism, hypothetically based on a conformational change upon melatonin binding, was supported using bulk‐solution evidence obtained by FRET, while qualitative structural rationale was further explored in silico through MD simulations. Bulk‐solution approaches such as FRET are widely regarded as valuable qualitative tools in the development of aptamer‐based biosensors, where the aptamer structure and redox‐reporter positioning are typically investigated before evaluating electrochemical surface responses [37]. Nevertheless, throughout this work, electrochemical behavior serves as the primary basis for comparison and interpretation.

The investigation was performed using two melatonin‐sensitive aptamer sequences named C1 and A2 (A2 is a truncated version of C1 [43]). FRET analysis was conducted using the fluorophore‐modified versions of C1 and A2 (Table S2) bearing Cy3 and Cy5 as donor and acceptor molecules, respectively. A specific concentration of the aptamer was added to different wells, and the emission of the acceptor fluorophore was recorded using a microplate reader (Figure 2a,b). Acceptor fluorescence arises from FRET, which depends on the donor–acceptor separation, and thus reports the aptamer's target‐induced conformational change. In the absence of the target, the aptamer adopts a compact fold that brings its labeled termini into close proximity, producing strong FRET (high acceptor emission). In the presence of melatonin, the aptamer is stabilized in a more structured state, placing Cy3 and Cy5 farther apart (Figure 2a,b), consistent with a drop in acceptor fluorescence. Indeed, this is one of the hypotheses that can be raised to explain why the intensity is decreased by increasing melatonin concentration. To verify this explanation, two different relatively high concentrations of the target were added to different wells of a plate containing the same concentration of the aptamer. Then, the emission of the acceptor fluorophore was recorded before and after the addition of the target at different time points. The results of these recordings have been presented in Figure 2c,d for C1 and A2 aptamers, respectively. The foremost observation is a concentration‐dependent decrease in emission following target addition. These observations support the explanation given above regarding the change in the distance between donor and acceptor molecules upon conformational change of the aptamer after interaction with the target. Another observation is the difference between the results of FRET experiments for C1 and A2 aptamers, when compared together. While both aptamers showed a decreasing response to increasing concentrations of melatonin, the change in the response of the A2 aptamer is more distinct (Figure 2d), implying a larger change in the distance between donor and acceptor molecules. Also, the absorbance peak of the acceptor molecule is lower than that of the donor molecule in the case of the C1 aptamer, both before and after the addition of the target (Figure 2c). However, for the A2 aptamer, the acceptor peak is larger than the donor's peak, and then it becomes smaller at high concentrations of melatonin. This distinct behavior suggests that, when aptamers are free in the solution, the A2 aptamer adopts a highly folded conformation that brings the fluorophore molecules into closer proximity. Upon target binding, a more pronounced conformational change occurs, leading to a sharper increase in the distance between the fluorophores. This explanation is in line with the linear structure of the A2 aptamer (Figure S1b), which can easily fold into itself and show more flexibility. However, the stem‐loop structure of the C1 aptamer (Figure S1a), which is less flexible, prevents the fluorophore molecules from being very close to each other before capturing the target and limits the change in their distance after target recognition. The effect of aptamer–target incubation time was further examined by measuring the emission signal at 10 and 20 min after target addition (Figure 2c,d; Figure S2a). There is no significant reduction in the emission signal from 10 to 20 min. This observation indicates that the interaction between the target and the aptamer reaches near completion within 10 min. While this test serves as a control suggesting that the recorded signal reaches saturation rather than continually decreasing, it also helps establish an appropriate incubation time between the target and aptamer for subsequent electrochemical experiments. We also tested a Cy3/Cy5‐labeled scrambled DNA sequence (Table S2) of the same length as the A2 aptamer to assess whether the FRET response is specific to the aptamer–target interaction. As shown in Figure S3b, the scrambled sequence exhibited a negligible normalized intensity change compared with the A2 aptamer. The corresponding normalized emission spectra are provided in Figure S2b,c. These results confirm the sequence dependence of the FRET signal and indicate that the observed response is not driven by nonspecific interactions between melatonin and the fluorophores.

**FIGURE 2:**
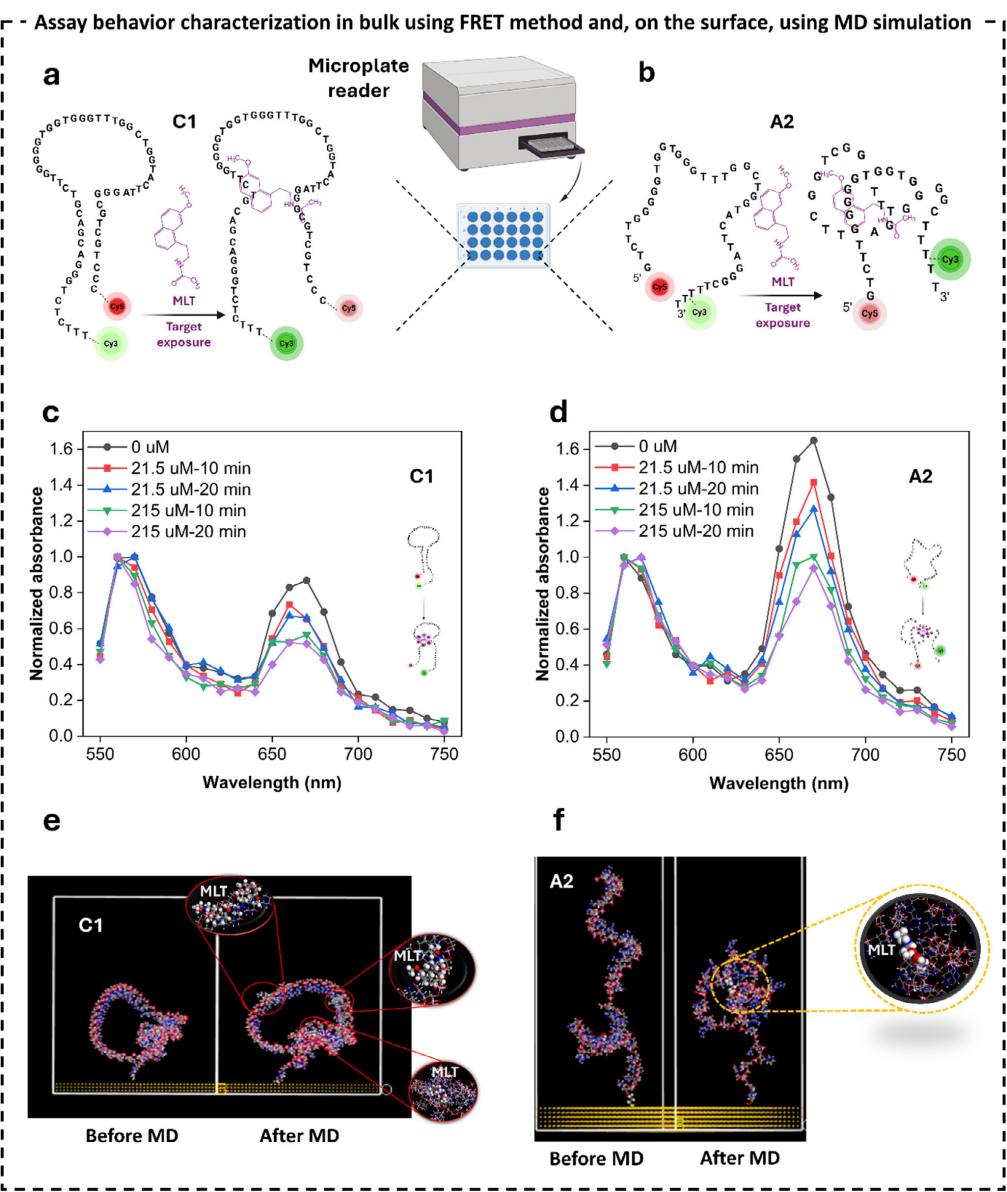
In bulk and simulation‐based qualitative investigations of the melatonin aptamer target‐responsive behavior and conformational change. (a) The experimental setup for FRET. (b) Schematic demonstration of hypothesized melatonin aptamer's conformational change upon exposure to the target in bulk solution. (c, d) Control FRET experiments to qualitatively support the target‐responsive behavior of the aptamer in bulk solution upon exposure to different concentrations of melatonin (c, d show the results for C1 and A2 aptamers, respectively). (e, f) MD simulation showing the conformational change hypothesized for the aptamer anchored onto the surface (e and f show the simulation for C1 and A2 aptamers, respectively).

The structural integrity and binding efficacy of C1, A2 aptamers, and the scrambled sequence were optimized through systematic geometry optimization (Figures S5b, S7b, and S9b). C1 aptamer exhibited the most stable optimization trajectory, reaching a refined global minimum with lower energy fluctuations compared to A2 and SS, suggesting superior structural rigidity post‐folding.

After that, MD simulations were performed using two independent grids (Box1 and Box2) for each aptamer, enabling the exploration of multiple potential binding regions on the target surface (Figures S5c,d, S7c,d, S9c,d). As shown in Figures S5e, S7e, and S9e, similar binding energy trends were observed across both docking boxes. C1 aptamer exhibited the strongest predicted binding affinity, with median docking energies around −5.0 kcal/mol and several conformers reaching below −6.0 kcal/mol, particularly in Box2. For A2, docking in Box2 yielded slightly stronger binding (median ≈ −4.7 kcal/mol) than in Box1 (≈ −4.0 kcal/mol), suggesting a preferred interaction region while maintaining favorable conformations in both grids. Among the candidates, the C1 aptamer showed comparable median energies in Box1 and Box2 (≈ −4.3 kcal/mol), although the energy distribution in Box2 was narrower (lower variability) than in Box1.

Further, MD simulations were performed to evaluate the structural stability and dynamic behavior of the aptamer–ligand complexes beyond the static docking poses. This approach enables assessment of conformational flexibility and the persistence of intermolecular interactions under near‐physiological conditions over time. MD snapshots extracted at 0%, 25%, 50%, 75%, and 100% of the 50‐ns trajectories were analyzed for all aptamers. For C1, the snapshots indicate an initial structural relaxation followed by the formation of a stable binding configuration. Although minor fluctuations are observed in peripheral loop regions, the core binding interface remains well preserved, suggesting stable and persistent interactions throughout the simulation (Figure S6). In the case of A2, the trajectory reveals an initial relaxation phase followed by a moderately stable binding state (Figure S8). While the peripheral loops are more flexible than those of C1, the core binding region remains preserved throughout the trajectory, indicating a dynamically stable yet more adaptable interaction during the simulation (Figure 2e,f). In contrast, MD snapshots for the SS aptamer show a progressive loss of interaction with melatonin over time. Although the ligand is initially positioned near the binding region, it gradually drifts away from the aptamer surface after the early stages of the trajectory. By the mid to late stages of the simulation, melatonin is largely displaced from the binding site, and no stable intermolecular interface is maintained. Together, these observations indicate that the SS–melatonin complex is dynamically unstable under the simulated conditions, unlike the more persistent interactions observed for the other aptamers (Figure S10).

After assessing each aptamer's conformational response to target binding in bulk solution (via FRET) and gaining qualitative insight into potential surface‐confined behavior through MD simulations, we systematically evaluated how aptamer sequence and MB reporter placement influence the performance of the electrochemical aptameric assay. This approach aligns with the overall focus of the study, which is to identify and optimize a melatonin‐responsive E‐aptasensor within established E‐AB frameworks, rather than to introduce a new transduction mechanism. Accordingly, we used three MB‐labeled variants of the C1 and A2 aptamers, with the reporter positioned at different loci along the sequence (Figure 3a–c). In the first two cases (Figure 3a,b), the C1 aptamer was modified with MB at its 3’ end or approximately in the middle of its sequence (C1m), while for the third case, the A2 aptamer was bearing MB at its 5’ end (Figure 3c). The mechanism of the electrochemical aptameric assay on the GWE surface has been shown in Figure 3a–c. SWVs were acquired over a series of melatonin concentrations, and the MB peak current was taken as the E‐aptasensor readout. The currents (normalized using a saturation‐based procedure based on Equation S1) for all three cases have been shown in Figure 3g (as well as three individual tests for each case in Figure 3d–f). As melatonin concentration increases, the MB peak current rises accordingly, consistent with a binding‐triggered conformational change that shortens the MB–electrode distance and accelerates electron transfer. The A2 aptamer, modified with MB at its 5′ end, exhibited the widest linear response across the tested concentration range compared to the other two cases. To better characterize and compare the surface behavior of these three aptamers, the HL binding model was fitted to their responses (Figure 3g) [44]. Using the HL binding model, the K_d, app_ was determined for each aptamer, with A2 exhibiting the lowest midpoint K_d, app_, corresponding to the most favorable apparent electrochemical response. To further characterize aptamer behavior, apparent electron‐transfer rates were estimated using the Laviron approach [39, 45]. In the absence of target, the estimated transfer rates for C1, C1m, and A2 were 164, 55, and 88 s^−1^, respectively (data not shown). Because A2 showed the most desirable sensing behavior, we also estimated its transfer rate at a saturating melatonin concentration to assess the effect of target binding on electron‐transfer kinetics; under these conditions, the apparent rate increased to 123 s^−1^ (data not shown). These values are consistent with prior studies of surface‐confined, MB‐modified aptamers [46]. Collectively, these results underscore differences in aptamer construction that likely contribute to their distinct electrochemical behaviors. They also support that melatonin binding modulates the apparent electron‐transfer rate, indicating that the concentration‐dependent signal changes originate from aptamer–target interactions rather than measurement artifacts. Moreover, previous work on electrochemical aptamer‐based biosensors has shown that binding kinetics and SWV parameters, particularly frequency, influence the apparent binding response [38, 47, 48, 49]. Accordingly, we optimized the SWV frequency for the A2 aptamer by maximizing its normalized response at a saturating melatonin concentration, identifying an optimum near 100 Hz (Figure S11).

**FIGURE 3:**
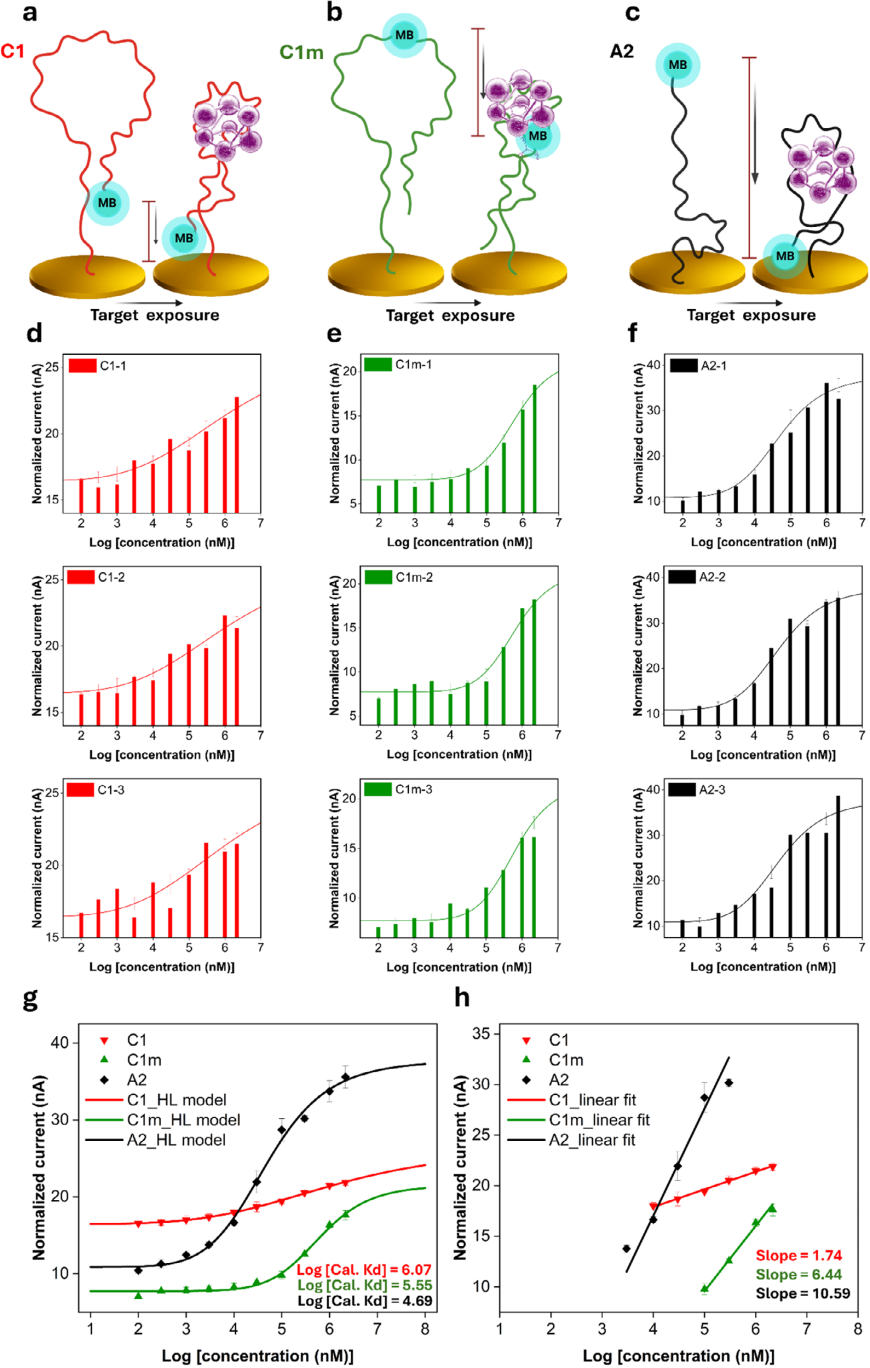
Performance characterization of the two different aptamer sequences (C1 and A2) against melatonin in the electrochemical conformational change‐based assay. (a–c) Schematic representation of aptamer conformational change upon target recognition for C1, C1m, and A2 aptamers. (d–f) Three individual tests performed using separate GWEs prepared with 100 nM of C1, C1m, and A2 aptamers, superimposed by their corresponding HL binding model fitted to their average normalized current. (g) HL binding model fitting on the average of data collected by the E‐aptasensors prepared with 100 nM of the C1, C1m, and A2 aptamers (the fittings were also overlaid on bar charts in (d–f)). R‐square for the HL fittings is 1, 0.99, and 0.99, respectively. (h) Linear fitting on the representative data around the inflection point of each binding curve shown in (g). Error bars are the standard error (SE) for three individual tests (*n* = 3).

It can also be speculated that the differing behaviors of these aptamers are attributable to their distinct pre‐binding secondary structures (Figure 3a–c). The A2 aptamer is largely linear before target exposure, giving the 5′‐MB label greater configurational freedom and a larger binding‐induced displacement (an essential determinant of signal in structure‐switching assays), which enhances distance‐dependent electron transfer. This yields higher sensitivity and a wider linear range (Figure 3c) [39, 50, 51, 52]. The A2 aptamer exhibits a greater capacity for target‐induced conformational change across concentrations than C1 or C1m. We hypothesize that A2's higher flexibility enables a larger binding‐driven reconfiguration, enhancing the distance‐dependent electrochemical signal. By contrast, the fixed stem–loop architectures of C1 and C1m likely restrict surface mobility and dampen signal transduction, yielding lower sensitivity relative to A2. Between the two stem–loop designs, C1m shows higher sensitivity than C1, consistent with its smaller K_d_ (i.e., higher apparent affinity). The reason for the higher sensitivity of C1m compared to C1 can be explained in the same way, considering the signal‐on behavior of the assay on the surface. The C1m aptamer has MB in the middle of its secondary structure (sequence), giving MB more movement capacity compared to MB in C1, which resides near the surface, having less room to move (Figure 3a,b). Because the C1 stem part is tethered to the electrode through one end of its stem, the duplex region is mechanically constrained and cannot undergo large conformational changes upon binding; this geometry limits the movement of the MB reporter on the other end of the stem and keeps it close to the surface. By contrast, C1m places MB on the more flexible loop farther from the surface and away from the tether, allowing greater conformational freedom upon target engagement and producing larger changes in reporter–electrode distance (and thus a stronger electrochemical modulation). This gives MB in the middle of the C1m aptamer more flexibility to move, making this aptamer more sensitive compared to C1. To better compare the sensitivities of these aptamers, linear fittings were done to the linear range of the binding curve of each aptamer (Figure 3h). In accordance with the previous discussion, the linear range of the A2 aptamer has the highest slope, showing its higher sensitivity, while this parameter for C1 is the lowest, suggesting its lower sensitivity (Figure 3h).

Overall, based on these observations, the differences in binding behavior among the aptamers most likely arise from a combination of interfacial chain dynamics, reporter placement, and target‐induced conformational reconfiguration, rather than being explained solely by a distance‐based mechanism, consistent with previous studies [53, 54].

Also, to show the consistency of the responses of these aptamers on the homemade GWEs, the three individual tests for each aptamer were shown in Figure 3d–f. In these figures, each plot is superimposed by the HL binding curve of the corresponding aptamer fitted to its average response of the three separate electrodes.

Since the A2 aptamer showed a superior response compared to C1 and C1m aptamers, this aptamer was used for subsequent optimization of the assay. Different immobilization conditions have been investigated for this aptamer to move the response range of the assay toward the physiological range of melatonin and achieve the highest sensitivity in this range. Our hypothesis for the optimization step is based on the premise that modulating the population or the surface loading of the detection probe (in this case, the MB‐modified aptamer) can shift the binding curve and signaling range of the molecular detection assay. Prior studies also support this optimization strategy as they show that the concentration of the recognition element can change the apparent response window of an assay [38, 55]. Figure 4a–d schematically depict the rationale behind the optimization study, showing how reducing the aptamer population can enhance sensitivity in the low concentration range of the target. The overall electrochemical signal (generated by the reduction of MB) of the aptameric biosensor is directly related to the aptamer population on the GWE surface. Decreasing the aptamer population on the GWE surface results in the subsequent reduction of the MB density on the surface, which in turn causes smaller MB peak currents (electrochemical signal) to be generated. On the other hand, at extremely low target concentrations, only a small fraction of the GWE‐immobilized aptamers binds and undergoes the conformational change (Figure 4a,c), resulting in subtle MB peak current change. We therefore hypothesize that the effect of aptamer population is more appropriately interpreted as a surface‐coverage–dependent interfacial effect, rather than simply a consequence of reduced absolute current. Accordingly, tuning the aptamer population, supported by the distinct packing densities estimated in Figure S12, alters surface interactions and the apparent transduction behavior of the surface‐confined aptamer assay. This, in turn, shifts the apparent response window of the E‐aptasensor (Figure 4a–d) [38, 49, 56, 57]. First, the effect of GWE size was investigated at a specific concentration and incubation time of the aptamer (Figure 4e,h). As expected, the peak currents measured using 4 mm diameter GWE were much higher compared to those for 1.5 mm diameter. Apart from a modest improvement in the apparent midpoint (K_d, app_), the overall behavior of the aptameric assay was unchanged. Based on this observation, the 1.5 mm diameter GWE was used afterward for all experiments, as the final fabricated platforms would be smaller and more cost‐effective. Next, the effect of aptamer immobilization time was investigated at a specific concentration by incubating the aptamer solution on the GWEs overnight (∼16 h) or for 1 h (Figure 4f,i). Again, the peak currents measured after overnight incubation were a little higher compared to those for 1 h, but no significant change in the overall behavior of the aptameric assay was observed. Therefore, all GWEs used in the subsequent experiments were incubated in the aptamer solution for 1 h, as this makes the preparation process of the sensing platform easier and more efficient. Then, the effect of aptamer solution concentration during the immobilization process was investigated. Different concentrations of the aptamer from 12 to 100 nM were used in this study. The results of the E‐aptasensors prepared with these concentrations are shown in Figure 4g,j. The first notable observation is that by reducing the aptamer concentration from 100 to 50 nM, the functional range of the aptameric assay does not significantly change, although the assay prepared at 100 nM showed a higher sensitivity. Reducing the aptamer concentration from 50 to 25 nM markedly shifted the functional range of the assay toward lower concentrations, aligning it with the physiological range of melatonin (Figure 4g,j). Further reduction of the aptamer concentration from 25 to 12 nM caused the aptameric assay to become unresponsive in the whole tested concentration range of the target. The reason for this observation is not that the assay is not working or the aptamers are not interacting with the target. This likely reflects potentiostat resolution limits, as the faradaic currents at this aptamer loading approach the instrument's noise/quantization floor. It should also be noted that as the concentration of the aptamer was reduced from 100 to 12 nM, the measured peak currents reduced respectively (Figure 4g). This aligns with the principle that reducing the aptamer concentration leads to a lower surface density of aptamers, resulting in fewer redox probes available to generate the electrical signal [38, 58]. This conclusion is supported by the fact that all biosensor preparation parameters, including the electrode substrate, immobilization time, and post‐passivation procedure, were kept constant, while only the aptamer concentration was varied during functionalization. This trend was further validated by integrating the MB SWV peaks to estimate the aptamer packing density for each immobilization concentration (Figure S12). The monotonic decrease in packing density observed in Figure S12 confirms that fewer MB‐modified aptamers were immobilized on the electrode surface as the aptamer concentration decreased. Moreover, the packing densities we obtained fall within the typical surface‐coverage range reported in the literature and remain below commonly reported saturation levels, indicating that the prepared aptamer monolayers are physically reasonable [38, 59]. For each parameter investigated in Figure 4, the individual result is shown separately in Figure 4e–g to better show the behavior of the aptameric assay under a certain condition. It should also be noted that the broad concentration span reported here reflects the combined response windows achieved using different aptamer surface populations, rather than the intrinsic dynamic range of a single fixed E‐aptasensor configuration [55, 60, 61, 62]. Overall, these results indicate that a low aptamer surface density is required to shift the apparent response window into the ultralow melatonin regime (approximately 1 pM–3 nM, encompassing the physiological range). Accordingly, an immobilization concentration of 25 nM (with a 1 h immobilization time) was selected for subsequent experiments as the most suitable configuration for measurements within the physiological range.

**FIGURE 4:**
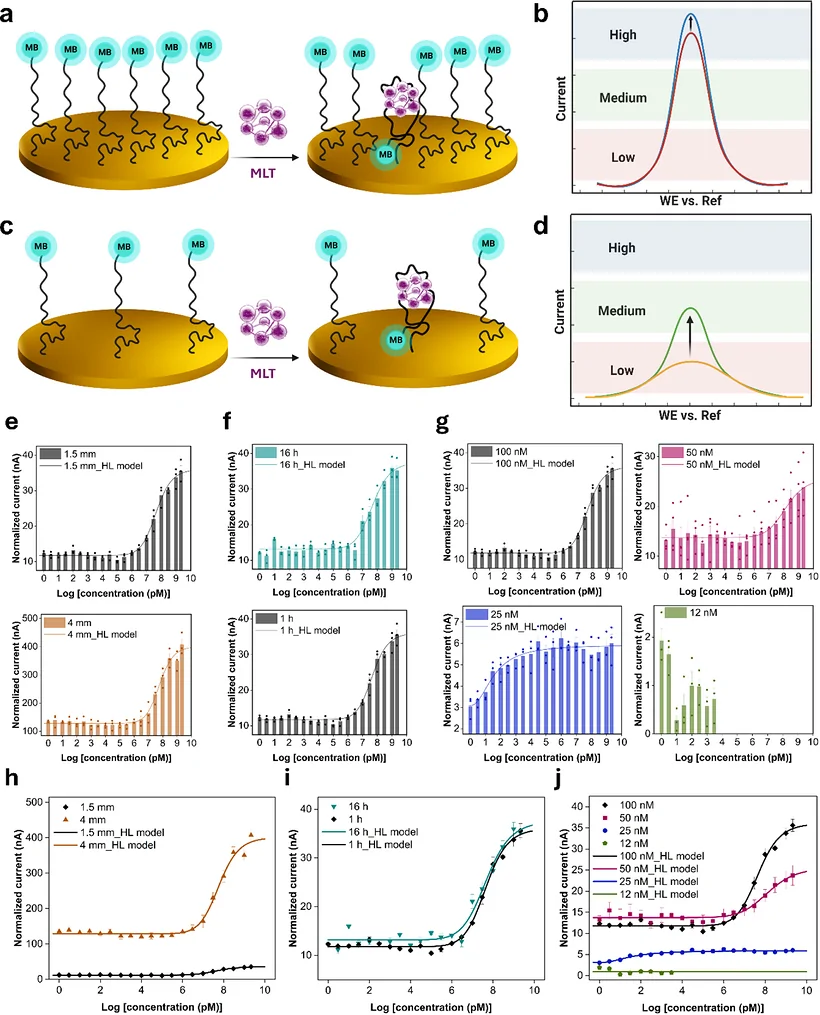
Aptameric assay optimization and the relevant effects on the E‐aptasensor performance. (a–d) Schematic demonstration of the aptamer population effects on the sensitivity. (e) Individual tests done using the A2 aptamer with two sizes of GWEs. (f) Individual tests done using the A2 aptamer with two different immobilization times. (g) Individual tests done using the A2 aptamer with four different concentrations of the aptamer (See the Supporting Information for packing density quantification). (h) Study on the effect of GWE size on A2 aptameric assay using the HL binding model. R‐square of the HL fitting is 0.99 for both. (i) Study on A2 aptamer immobilization time using the HL binding model. R‐square of the HL fittings is 0.96 and 0.99, respectively. (j) Study on A2 aptamer population by varying concentrations and the HL binding model for each concentration, comparing response windows of E‐aptasensors prepared with different aptamer loadings (see Figure S12 for packing density quantification). R‐square of the HL fittings are 0.99, 0.95, and 0.94, respectively. Error bars are the SE for three individual tests (*n* = 3).

After optimizing the assay parameters and shifting its responsive range toward the physiological range of melatonin, the analytical performance of the aptameric assay was investigated. Figure 5a schematically illustrates the A2 aptamer‐based assay. It should be emphasized that the sensing chemistry is direct, one‐step, and reagentless, meaning that the pre‐functionalized electrode is simply exposed to the target solution and subsequently measured electrochemically. However, for development‐stage characterization and normalization, an additional saturation measurement was performed at a high melatonin concentration. Based on the optimization results presented in Figure 4, an aptamer immobilization concentration of 25 nM was selected for subsequent analytical studies, as it produced a significantly reduced but still quantifiable surface coverage (Figure S12) while shifting the sensor's response window toward the physiological melatonin concentration range. Figure 5b represents the normalized current of the aptameric assay prepared using a 25 nM concentration of the aptamer for target concentrations less than 3 nM, covering the physiological range of melatonin. The response of the assay is overlaid with the HL binding model, which has a K_d, app_ of 37 pM. The inset of this plot is the linear fitting on the representative data around the inflection point of the binding curve, showing a sensitivity of 0.98 and a LOD of 0.75 pM.

**FIGURE 5:**
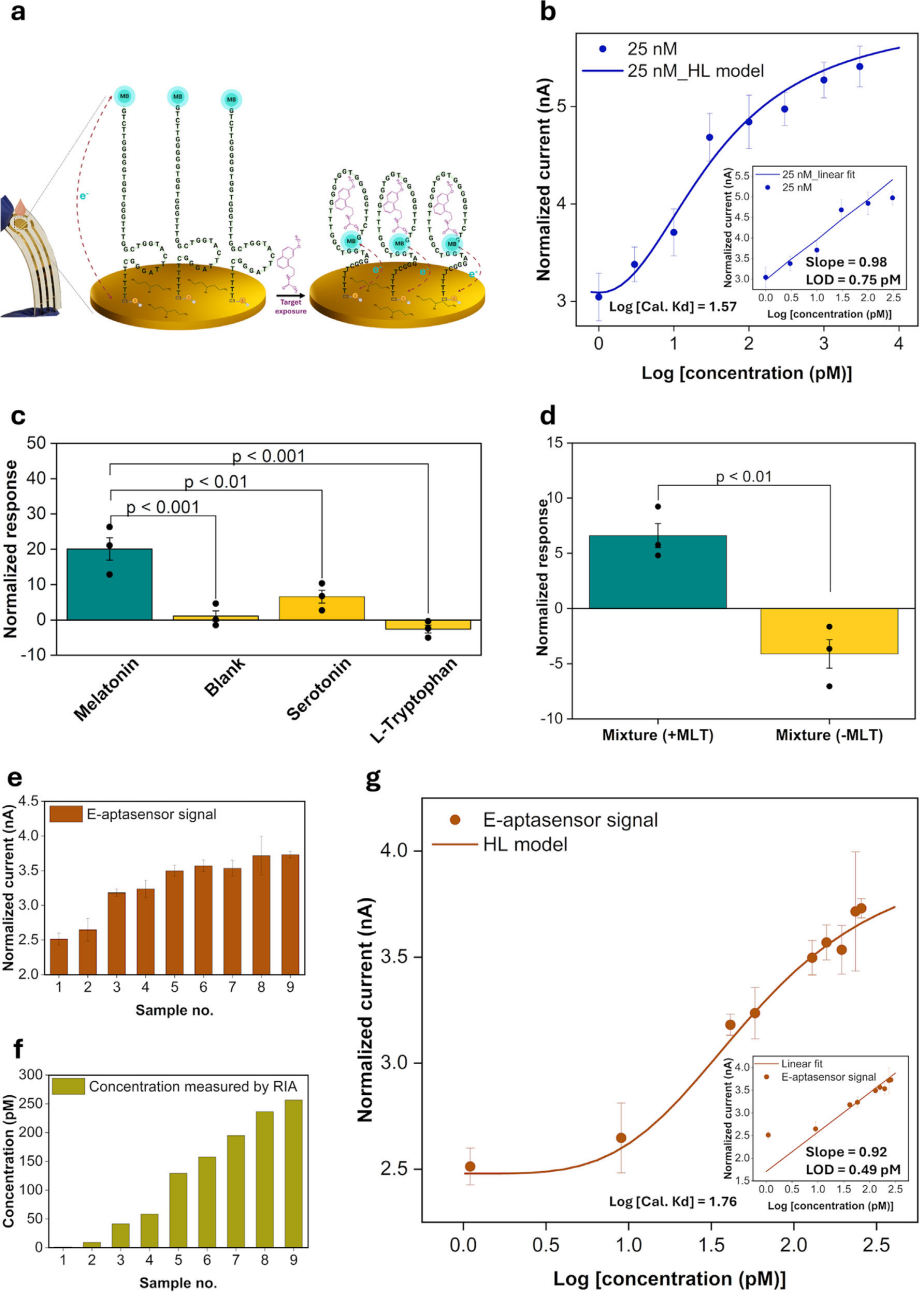
Analytical performance. (a) Schematic demonstration of the aptameric assay. (b) Normalized currents and HL binding model (R‐square = 0.94) at physiological range (≈ 1–300 pM) for 25 nM population of A2 aptamer (the inset of the plot is the linear fitting on the representative data around the inflection point of the binding curve). (c, d) Individual and mixture‐based interference studies using a targeted panel of relevant melatonin interferents. (e) Normalized currents of the A2 aptameric assay directly tested with 9 untreated human plasma samples. (f) Concentration of the 9 untreated human plasma samples measured using RIA. (g) HL binding model fitted (R‐square = 0.99) to the calibration curve directly obtained in characterized untreated human plasma samples for a 50 nM population of A2 aptamer (the inset of the plot is the linear fitting on the representative data around the inflection point of the binding curve). Error bars are the SE for three individual tests (*n* = 3).

Next, the selectivity of the aptameric assay was evaluated both individually and in mixtures against a targeted panel of relevant potential interferents. Although serotonin produced a measurable response when tested alone, mixtures containing melatonin yielded a clearly distinguishable response compared with the corresponding interferent mixture without melatonin (Figure 5c,d). This demonstrates the practical selectivity and discriminatory capability of the assay under the tested conditions and is further supported by sequence‐specific controls (Figure S3). To further confirm that the signal arises from a specific aptamer–target interaction, we tested an MB‐modified scrambled DNA sequence (Table S2 and Figure S1c) of the same length as the A2 aptamer. As shown in Figure S3a, this randomly selected sequence produced a negligible normalized response compared with the A2 aptamer.

These results support that the electrochemical signal is primarily generated by the specific A2–melatonin interaction, rather than by a direct effect of melatonin on MB or nonspecific interactions at the electrode surface. A key step in translational implementation of an aptasensor is its acceptable performance in complex biological matrices, as it has been demonstrated in prior studies [39, 41]. To assess performance in a clinical matrix and establish a plasma‐based calibration for future clinical studies, we analyzed 12 unextracted human plasma samples with RIA‐assigned concentrations (Table S3). Figure 5e shows the mean normalized currents obtained from independently prepared GWEs, which were generally measured in triplicate when sufficient sample volume was available. Among the 12 unextracted human plasma samples characterized by RIA, nine were included in the primary analysis (Figure 5e–g), while three were excluded because electrode‐specific variability resulted in response patterns that were inconsistent with the principal response window (see Figure S13). RIA‐assigned concentrations for these samples are shown in Figure 5f. Together with the aptasensor responses, the data demonstrate a dose‐dependent increase in unextracted human plasma, confirming that the assay reliably tracks melatonin across concentrations. To better characterize the assay behavior in human plasma, a response signal plot (Figure 5g) was made for the plasma samples based on the normalized currents shown in Figure 5e and the concentrations depicted in Figure 5f. The HL binding model that was fitted to the response in Figure 5g has a K_d, app_ equal to 57 pM. The inset of this plot is the linear fitting on the representative data around the inflection point of the binding curve, showing a sensitivity of 0.92 and a LOD of 0.49 pM. Importantly, the reported K_d, app_ values should not be directly compared with the solution‐phase K_d_ of the corresponding aptamer, as these values represent apparent, sensor‐dependent parameters that reflect the behavior of surface‐confined MB‐modified aptamers under specific surface preparation and electrochemical interrogation conditions [38, 63, 64, 65].

Compared with buffer, assay performance in human plasma shows a modest decline, as reflected by the higher apparent midpoint observed in plasma. This shift is attributable, at least in part, to the higher aptamer concentration (50 nM instead of 25 nM) used during electrode immobilization for patient testing. Electrodes that were prepared with a 25 nM aptamer concentration for the experiments in plasma did not produce repeatable measurable signals, as they were more sensitive to the electrode‐to‐electrode variations. However, the assay performance in untreated plasma remains acceptable, as the sensor clearly distinguishes among the melatonin concentrations tested. Importantly, this performance was achieved directly in unextracted plasma (a key differentiating feature highlighted in Table S1), whereas RIA requires a prior extraction step. The primary plasma analysis in this study is the direct, matrix‐matched calibration in untreated human plasma using samples with melatonin concentrations pre‐characterized by RIA (Figure 5e–g). Our conclusion that the developed E‐aptasensor can detect melatonin in untreated plasma is based on this matrix‐matched analysis. We also provide an exploratory comparison of plasma and buffer calibration curves in the Supporting Information (Figure S14); however, this does not represent the analytical workflow and is included only as a secondary assessment.

It should also be noted that a series of experiments was conducted to evaluate the effects of storage conditions, temperature, and measurement media on the robustness of the E‐aptasensor response. Although a decline in baseline current was observed for E‐aptasensors stored at 4°C, 25°C, and 37°C (Figure S15a), the sensors retained most of their signal‐generation capability after 10 days of storage under these conditions (Figure S15b). As shown in Figure S15b, E‐aptasensors stored at 37°C exhibited the most significant loss in response, consistent with previous reports demonstrating accelerated degradation of aptasensor sensing layers at elevated temperatures compared with refrigerated or room‐temperature conditions [45, 66].

The E‐aptasensor was also evaluated in artificial serum and ISF with different viscosities at various temperatures. Under these conditions, the E‐aptasensor remained operational, although its normalized response was slightly attenuated, particularly at higher temperatures (Figure S15c). These results demonstrate acceptable performance across different matrices despite some signal attenuation, in agreement with previous studies [47, 67].

Finally, cyclic voltammetry (CV) was performed to examine MB peak currents as a function of scan rate. As shown in Figure S15d, the MB peak current increased with increasing scan rate, as expected for a surface‐confined redox species and consistent with prior studies of MB‐modified electrochemical platforms [68]. Notably, these CV data were acquired using a commercial portable potentiostat, further highlighting the potential of the E‐aptasensor for future translational and point‐of‐care applications.

## 3 Conclusion

Current clinical diagnosis and monitoring of circadian misalignment and related conditions (e.g., CRSDs) still rely largely on nonspecific proxies such as sleep logs and actigraphy. Although melatonin profiling is the gold standard, it requires serial sampling with stringent storage and processing at logistically inconvenient and variable time points. We demonstrated and clinically challenged an E‐aptasensor built on established electrochemical aptamer‐sensing frameworks, rather than introducing a new transduction principle. This platform quantifies melatonin directly in unextracted plasma using a direct, reagentless electrochemical readout, with no sample‐preparation step. By engineering the sensing layer, including selecting a structure‐switching melatonin aptamer (A2), positioning the MB reporter, and tuning aptamer surface population, the platform achieved apparent picomolar sensitivity across the physiological range (sub‐pM LODs in buffer and plasma) and practical discrimination of melatonin against a targeted panel of relevant interferents. Additionally, mechanistic validation by FRET and molecular modeling supported the structure‐switching assay principle. Notably, based on calibration against RIA‐characterized untreated samples, the E‐aptasensor quantified melatonin directly in unextracted plasma with acceptable accuracy, thereby avoiding the extraction step typically required for standard immunoassays (e.g., ELISA and RIA). Brief stability experiments further provided preliminary but promising evidence of the E‐aptasensor's practical robustness, with substantial retention of function across the tested conditions. Collectively, these results support the clinical feasibility of future workflows for rapid, time‐resolved melatonin profiling without extraction, positioning the E‐aptasensor as a promising complement to gold‐standard laboratory immunoassays. Therefore, as an application‐focused advance, this study extends the E‐AB framework to enable the detection of physiological‐range melatonin directly in untreated plasma. In addition, preliminary tests demonstrated compatibility with portable instrumentation, including a commercial portable potentiostat; however, full system integration and validation for point‐of‐care use remain the subject of future work. Moreover, this E‐aptasensor was evaluated in a single‐use, single‐electrode‐per‐sample format. Accordingly, sensor regeneration was not investigated in this work and will be explored in future studies. Although the sensing chemistry is direct and reagentless, this study uses normalization to correct electrode‐to‐electrode variability. Accordingly, this normalization is best viewed as a development‐stage correction within a proof‐of‐concept workflow that will be refined as the platform advances toward a standardized, ready‐to‐use clinical format. Future work will prioritize reducing batch‐to‐batch variability in both the electrodes and the sensing layer and implementing improved baseline‐correction/normalization strategies to mitigate the observed discrepancies. In parallel, we will further advance this platform for point‐of‐care and at‐home testing to enable ambulatory, time‐resolved phase typing and longitudinal monitoring of CRSDs and circadian disruption.

## Author Contributions

**Hamed Shieh**: Conceptualization, Data curation, Formal analysis, Investigation, Methodology, Validation, Visualization, Writing – original draft, Writing – review & editing. **Arash Khorrami Jahromi**: Conceptualization, Data curation, Formal analysis, Investigation, Methodology, Validation, Visualization, Writing – original draft, Writing – review & editing. **Fernando Gonzales‐Aste**: Conceptualization, Investigation, Writing – original draft, Writing – review & editing, Resources. **Sajad Shiekh**: Investigation, Methodology, Validation, Visualization, Writing – original draft. **Roozbeh Siavash Moakhar**: Software, Methodology, Validation, Visualization, Writing – original draft. **Sahar Sadat Mahshid**: Conceptualization, Methodology, Writing – review & editing, Project administration, Supervision. **Sara Mahshid**: Conceptualization, Funding acquisition, Project administration, Supervision, Writing – review & editing.

## Conflicts of Interest

The authors declare no conflicts of interest.

## Supporting information




**Supporting File**: advs76882‐sup‐0001‐SuppMat.docx.

## Data Availability

The data that support the findings of this study are available from the corresponding author upon reasonable request.
