# Supplementary material for: Single‐Step Electrochemical Aptasensor for Melatonin Detection in Unprocessed Plasma
Source: Adv Sci (Weinh). 2026 Nov 4:e76882. Online ahead of print. doi: 10.1002/advs.76882 (PMC13736177; doi:10.1002/advs.76882)
Supplement: Supplementary file 1 [file ADVS-9999-e76882-s001.docx]

**Supporting Information**

**for**

**Single-Step Electrochemical Aptasensor for Melatonin Detection in Unprocessed Plasma**

Hamed Shieh^a^, Arash Khorrami Jahromi^a^, Fernando Gonzales-Aste^b^, Sajad Shiekh^a,c^, Roozbeh Siavash Moakhar^a^, Sahar Sadat Mahshid^d*^, Sara Mahshid^a,e*^

[a] Department of Bioengineering, McGill University, Montreal, Quebec, Canada

[b] Department of Psychiatry, Faculty of Medicine, McGill University, Montreal, Quebec, Canada

[c] Department of Physics, McGill University, Montreal, Quebec, Canada

[d] Beeta Biomed, Clinical Innovation Platform, Montreal General Hospital, Montreal, Quebec, Canada

[e] Division of Experimental Medicine, McGill University, Montreal, Quebec, Canada

**Methods**

Materials and reagents

Melatonin, serotonin, tryptophan, 6-Mercapto-1-hexanol (MCH), Tris-(2-carboxyethyl)-phosphine hydrochloride (TCEP), magnesium chloride (MgCl_2_), and phosphate-buffered saline (PBS) were purchased from Sigma-Aldrich. Artificial ISF and serum were purchased from Biochemazone. Medical-grade pressure-sensitive adhesives (PSA) were purchased from Adhesives Research, and self-adhesive removable vinyl films were purchased from BDF Graphics. Acrylic films were purchased from McMaster-Carr. Screen-printed gold electrodes (SPGEs) were purchased from Metrohm DropSens. The Ag/AgCl reference electrode and platinum wire counter electrode were purchased from PalmSens. Modified aptamers used in the study were purchased from Integrated DNA Technologies (IDT). **Table S2** shows the sequences of these aptamers.

Fabrication of the electrode platforms

The homemade GWEs were fabricated on acrylic films using stencil lithography. More details of the fabrication process can be found in the SI (**Fig. S17**). Briefly, the stencil (vinyl adhesive film with 0.5 mm thickness) was attached to the acrylic film. Then, the GWE design was cut into the stencil by a cutting plotter device. Later, the GWE was made by first depositing 20 nm of chromium as the adhesion layer and then 100 nm of gold using DC sputtering. Finally, the stencil was removed and the whole electrode, except the connection pads and the working area, was covered with PSA.

Biosensor preparation

All GWEs were washed with copious amounts of DI water before functionalization. PBS buffer solution (1X PBS with 2 mM MgCl_2_) was used in all steps of this study. The DNA aptamer sequences were heated at 95 °C for 5 minutes in a solution containing 50 uM of the aptamer in PBS and gradually cooled to room temperature to help them fold into their secondary structure. Then, the folded aptamer sequences were reduced for 2 h at room temperature in the dark in a solution containing 25 uM of the aptamer and 5 mM of TCEP in PBS. Immediately after being reduced, the aptamer solution was diluted to the desired concentration using PBS and then applied to the working area of the GWEs. The GWEs were incubated with the aptamer solution for 1 h or overnight at room temperature in a dark, humid chamber and then washed with PBS. Finally, the aptamer-modified GWEs were incubated with a solution of 3 mM MCH in PBS for 3 h again at room temperature in a dark, humid chamber to block the unbound areas of the GWEs and remove any weakly attached aptamer. After that, the GWEs were washed with PBS before using them for electrochemical measurements.

Electrochemical measurements

SWV technique was used to perform all electrochemical measurements using an AutoLab potentiostat. All measurements were done at room temperature. SWV was performed in a potential range of 0 V to –0.6 V and an amplitude of 0.025 V at a frequency of 100 Hz. The apparent electron transfer rates were determined using Laviron method by running CV scans in the range of 0.025 to 50 V/s [1, 2]. All electrochemical measurements were performed using a standard three-electrode system with Ag/AgCl reference electrode and a platinum wire counter electrode. The same system was used for running CV measurements using a PalmSens portable potentiostat between 0 V and -0.5 V at scan rates in the range of 0.01 to 0.4 V/s. For CV measurements, three scans were done and the results were extracted from the average of the last two scans. The packing densities were estimated from the integrated charge under the methylene blue SWV peak [3-5], normalized to the geometric surface area of the GWE. All measurements were performed in the target solution after 10 minutes of incubation on the biosensor surface. After reading the target signal (the SWV peak current), the target solution was replaced by a solution of a saturating concentration of melatonin, which ensures that all active aptamers on the biosensor surface interact with the target. This saturation-signal measurement is used solely as part of the development-stage normalization/characterization workflow and is performed within each aptamer population condition; therefore, it does not mathematically enforce similar normalized saturation responses across different surface populations. After 10 minutes of incubation, another SWV scan was conducted in this high-concentration solution to determine the saturation signal of each biosensor.

For each condition in this study, we normalized the current of each biosensor using **Eq. S1** by comparing its saturation current to the mean saturation current of all electrodes prepared with the same aptamer population. This converts the measured current to a saturation-corrected current referenced to the average saturation signal for that population, thereby reducing (but not fully eliminating) batch-to-batch variability in electrode surfaces during calibration. Notably, this normalization approach differs from the common baseline normalization that uses the signal in the absence of target.

For selectivity, stability, and control experiments, we instead reported the percentage normalized response using **Eq. S2**, which is more suitable for comparing responses across conditions.

$$Normalized current=Current_{t}\times\frac{Current_{\frac{sat}{ave}}}{Current_{sat}} Eq.S1$$

$$Normalized response=100\times\frac{(Current_{t}-Current_{0})}{Current_{0}} Eq.S2$$

Where $Current_{t}$ is the target current of each biosensor, $Current_{sat}$ and $Current_{0}$ are the saturation and baseline currents of the same biosensor, and $Current_{sat/ave}$ is the mean saturation current of all biosensors prepared with the same aptamer population as the biosensor being tested.

FRET measurements

Aptamers were diluted from 100 µM to a final concentration of 2 µM in 100 µL of PBS. MLT was titrated sequentially into the DNA-containing wells over a concentration range of 21.5 uM to 215 uM, with the added volume adjusted to achieve the desired final concentrations. Control samples containing aptamer only and buffer alone were monitored over time, and no significant changes in signal were observed under these conditions (**Fig. S2a**). FRET measurements were conducted using a SpectraMax plate reader in a 96-well format, with excitation at 540 nm and emission spectra collected from 550 nm to 750 nm. The emission data was normalized with respect to Cy3 emission peak at 560 nm.

Molecular modeling and simulation

The structural and interaction analysis of the aptamer-ligand complex was conducted through a multi-stage computational approach. Initially, the three-dimensional structure of the aptamer was predicted from its nucleotide sequence using thermodynamic ensemble modeling at 25 °C under standard ionic conditions. Molecular geometry optimization of both the aptamer and the ligand was performed using the Universal Force Field (UFF) with 3000 iterations to ensure structural stability. Docking studies were carried out using AutoDock4, employing a genetic algorithm with 100 runs. The docking grid was centered at coordinates (13, −25, −13) with box dimensions of (126, 108, 114), and the resulting binding energies ranged from −5.9 to −4.3 kcal/mol. The top-ranked binding conformers were then subjected to further optimization using the COMPASSIII Force Field for 80 iterations. Subsequently, molecular dynamics (MD) simulations were performed using the Desmond software package in a TIP3P water model under periodic boundary conditions over 50 nanoseconds to mimic physiological conditions. The root mean square deviation (RMSD) profiles qualitatively supported the structural stability of the aptamer-ligand complex, with the aptamer stabilizing in the 12–13 Å range and the ligand consistently bound with an RMSD of 1.5–2.5 Å. Visualization of MD snapshots qualitatively supported the persistence of non-covalent interactions and overall structural integrity throughout the simulation period. However, it should be noted that these data do not directly represent the dynamics of the immobilized aptamer under electrochemical interrogation.

Clinical analysis

Blood samples from healthy participants were collected and shared de-identified by our collaborators from the Mass General Brigham Institution/Brigham and Women's Hospital, in Boston, USA. Samples were collected under the approval of Mass General Brigham Institutional Review Board (Protocol 2019-P-001247). Samples were collected during a constant routine (CR) protocol and measured for their content of melatonin using RIA by Solidphase. In a typical constant routine designed to assess endogenous circadian rhythms, such as melatonin, participants remain awake in a controlled lab environment for ~24–40 hours under strictly constant conditions to minimize behavioral masking. Posture is kept supine or semi-recumbent, with minimal movement; ambient light is dim (often <10 lux) and without time cues (no clocks, phones, or windows). Temperature is thermoneutral and stable. Small, isocaloric snacks and water are provided at regular intervals. Caffeine, alcohol, nicotine, and medications affecting sleep or the circadian system are prohibited for a washout period beforehand and during the CR. Blood samples are collected at fixed intervals for melatonin assay. This protocol allows the unmasked circadian profile of melatonin (e.g., onset, peak, amplitude, and timing) to be estimated with minimal influence from sleep, posture changes, activity, meals, or light.

**Table S1.** Comparison of conventional melatonin assays and recently reported melatonin sensing platforms.

| **Method/platform** | **Recognition element** | **LOD** | **Detection/working range** | **Extraction required** | **Readout time** | **Application matrix** | **Reference** |
| --- | --- | --- | --- | --- | --- | --- | --- |
| Conventional RIA | Antibody | Kit-/lab-dependent | Kit-/lab-dependent | Often required | Hours | Plasma, serum, saliva | [6, 7] |
| Conventional ELISA | Antibody | Kit-dependent | Kit-dependent | Often required | Hours | Saliva, plasma, serum | [6, 7] |
| Voltammetric sensor | MIP | 29 nM | 0.097 to 200 µM | NR | NR | Saliva, urine | [8] |
| Voltammetric sensor | MIP | 0.171 µM | 0 to 100 µM | NR | 10 min | Standard solution | [9] |
| Colorimetric AuNP assay | DNA aptamer | 0.0011 nM | 0.005 to 500 nM | NR | NR | Saliva | [10] |
| Voltammetric sensor | MIP | 0.027 µM | 1 to 1000 µM | NR | NR | Serum | [11] |
| Competitive ELAA | DNA aptamer | 2.5 pM | 39 pM to 8.62 µM | NR | ≥2.5 h | Saliva | [12] |
| Voltammetric sensor | Direct detection on BDD | 55.5 nM | 1 to 7 mM | NR | NR | Liver tissue matrix | [13] |
| EIS | Antibody | 0.15 pg/mL | 0.2 to 72.9 pg/mL | NR | NR | Sweat | [14] |
| SiNW-FET | DNA aptamer | fg/mL level | 15 fg/mL to 1.5 ng/mL | Not reported | 2 min | Saliva | [15] |
| Ratiometric electrochemical sensor | MIP | 3.78 nM | 5 nM to 50 μM | NR | NR | Saliva, urine, serum, plasma, ISF | [16] |
| This work | DNA aptamer | 0.49 pM | ~1 pM to 3 nM | No | 10 min | Plasma | This work |

**Abbreviations:** AuNP, gold nanoparticle; ELAA, enzyme-linked aptamer assay; FET, field-effect transistor; ISF, interstitial fluid; MB, methylene blue; MIP, molecularly imprinted polymer; BDD, boron-doped diamond; NR, not clearly reported.

**Table S2.** Sequences of the aptamers used in this study.

| **Electrochemistry** | **Sequence 5′ to 3′** |
| --- | --- |
| C1 | 5'- /5ThioMC6-D/ TTT CTC TCG GGA CGA CGT CTT GGG GGT GGT GGG TTT GGC TGG TAC TTA GGG CGT CGT CCC /3MeBIN/ -3’ |
| C1m | 5'- /5ThioMC6-D/ CTC TCG GGA CGA CGT CTT GGG GGT GGT GGG /iMeBIN/TT TGG CTG GTA CTT AGG GCG TCG TCC C -3' |
| A2 | 5'- /5MeBIN/ GTC TTG GGG GTG GTG GGT TTG GCT GGT ACT TAG GGC TTT TT/3ThioMC3-D/ -3' |
| Sqr. Seq. | 5'- /5ThioMC6-D/ TTT TTT ACT GCA GCG ATT CTT GTT TCC GAC CGG CAG CGG GC / 3MeBIN/ -3' |
| **FRET** |  |
| C1 | 5'- /5Cy3/ TTT CTC TCG GGA CGA CGT CTT GGG GGT GGT GGG TTT GGC TGG TAC TTA GGG CGT CGT CCC /3Cy5Sp/ -3' |
| A2 | 5'- /5Cy5/ GTC TTG GGG GTG GTG GGT TTG GCT GGT ACT TAG GGC TTT /iCy3/T T -3' |
| Sqr. Seq. | 5'- / 5Cy5 / TAC TGC AGC GAT TCT TGT TTC CGA CCG GCA GCG GGC / 3Cy3 / -3' |

*MeB: methylene blue

**Figure S1.** Structures of the aptamer sequences used in this study predicted by UNAFold web server. a) C1 aptamer, b) A2 aptamer, and c) scrambled sequence DNA with same length as A2 aptamer.

**Figure S2**. a) Normalized absorbance of control samples containing aptamer only and buffer alone monitored over time for C1 and A2 aptamers. b & c) Control FRET experiments to investigate the conformational change of A2 aptamer and the scrambled sequence in bulk solution upon exposure to different concentrations of melatonin (b and c show the results for A2 aptamer and the scrambled sequence, respectively).

**Figure S3.** a) Electrochemical responses of MB-modified A2 aptamer and a scrambled sequence of the same length to a saturating melatonin concentration. b) Normalized Cy5/Cy3 intensity changes for the labelled A2 aptamer and the corresponding scrambled sequence upon exposure to two melatonin concentrations in the FRET experiment.

**Molecular dynamics simulation**

The molecular dynamics (MD) simulation was conducted using the Desmond software package to evaluate the structural stability and binding behavior of the aptamer-ligand complex. The system was solvated in a TIP3P water model and simulated under periodic boundary conditions for a total duration of 50 nanoseconds. This simulation aimed to mimic physiological conditions and assess the dynamic interactions between the aptamer and its target ligand in an explicit aqueous environment.

**Figure S4.** Melatonin definition in the docking software. a) Melatonin chemical structure, and b) optimization configuration.

**Figure S5.** C1 aptamer definition in the docking software. a) Predicted 3D structure of designed aptamer. b) Energy minimization curve resulting from structural optimization. c) Docking location selection (box1). d) Docking location selection (box2). e) The variation of the binding energy of all molecular conformers.

**Figure S6.** Representative MD snapshots (0%, 25%, 50%, 75% and 100% of the 50-ns trajectory) indicate that the C1 aptamer preserves its global fold while exhibiting expected flexibility in peripheral/loop regions.

**Figure S7.** A2 aptamer definition in the docking software and its docking prediction. a) Predicted 3D structure of designed aptamer. b) Energy minimization curve resulting from structural optimization. c) Docking location selection (Box1). d) Docking location selection (Box2). e) The variation of binding energy of all molecular conformers.

**Figure S8.** Representative MD snapshots (0%, 25%, 50%, 75%, and 100% of the 50-ns trajectory) indicate that the A2 aptamer preserves its global fold while exhibiting expected flexibility in peripheral/loop regions.

**Figure S9.** Scrambled sequence DNA definition in the docking software. a) Predicted 3D structure of designed aptamer. b) Energy minimization curve resulting from structural optimization. c) Docking location selection (Box1). d) Docking location selection (Box2). e) The variation of binding energy of all molecular conformers for each Box.

**Figure S10.** Representative MD snapshots (0%, 25%, 50%, 75%, and 100% of the 50-ns trajectory) indicate that the aptamer (scrambled sequence) preserves its global fold while exhibiting expected flexibility in peripheral/loop regions.

**Figure S11.** a) Laviron plots for the A2 aptasensor constructed from the anodic (Ep,a) and cathodic (Ep,c) methylene blue peak potentials obtained by CV at varying scan rates. b) Normalized response of the A2 aptasensor to the saturating concentration of melatonin obtained at different SWV frequencies.

**Figure S12.** Packing density of the A2 aptamer on the electrode surface for sensors prepared using 25, 50, or 100 nM of aptamer.

Here, each aptamer concentration used in the immobilization stage produced a systematically different electroactive surface population, while all other parameters were kept the same during functionalization. Accordingly, the differences in the response windows observed in **Fig. 4** can be interpreted in terms of changes in surface populations and associated interfacial interactions of the MB-modified aptamers. Moreover, the packing density values in **Fig. S12** fall within the typical range reported in the literature and remain below commonly reported saturation levels [17, 18].

**Fig. S13** shows multiple test repetitions of each characterized human plasma sample used in this study. For each repetition, shown as a black dot, a single separate GWE was used. The number of repetitions for each sample depends on how much volume was available for running the tests. The variability between different repetitions of each sample comes from the difference in the population of the aptamers on the surface of the corresponding GWEs that were used. These observations indicate that, while saturation-based normalization is partially effective, it does not fully eliminate electrode-to-electrode variability. They also highlight a key limitation of the current platform: variations in sensing-layer population can shift the apparent response window. Accordingly, the exclusion of certain measurements in this study should be viewed not as a feature of a finalized analytical workflow, but as part of a development-stage data curation process. Based on the optimization process, we showed that the population of aptamers on the GWE surface has a direct effect on the responsive range of the assay. Because of the variability in aptamer population, each GWE might respond to a different concentration range—even though all are technically working. In other words, each electrode, being technically functional, might respond to a different concentration range due to the variability in aptamer population. Some GWEs are more sensitive to lower levels (which is desired here), and others to higher levels. **Fig. S13** also shows three typical response patterns (binding curves). Two of them (upper purple and bottom yellow) respond to higher concentrations (likely from GWEs with a high population of aptamers). The middle curve represents the optimal response pattern—sensitive to lower, more relevant concentrations. This curve closely matches the one found in earlier optimization experiments, confirming it's the most suitable for clinical use. The brown and yellow bars in **Fig. S13** are the average current of the repetitions that follow one of the three binding curves. For the upper binding curve (purple), error bars are not shown because there were insufficient data points in that range. The cyan points indicate samples whose responses could not be confidently assigned to one of the three principal response patterns and were therefore excluded from the primary analysis. This behavior is consistent with electrode-to-electrode variability in aptamer population and highlights a limitation of the current platform, despite the use of saturation-based normalization. Also, it should be noted that, although the current level of replication is sufficient for establishing proof-of-concept analytical behavior, these observations highlight the limited robustness of the current workflow and create motivation for future development of standardized protocols for data curation and sensing layer formation.

**Figure S13**. Test repetitions for human plasma samples and their corresponding binding curves.

The primary plasma analysis in this study is the direct, matrix-matched calibration in untreated human plasma using samples with melatonin concentrations pre-characterized by RIA (**Figs. 5e–g**). Our conclusion that the E-aptasensor can detect melatonin in untreated plasma is based on this analysis. We also include an exploratory comparison of plasma and buffer calibration curves; however, this does not represent the analytical workflow and is provided only as a secondary assessment. Specifically, **Fig. S14** is intended to illustrate how the calibration curves relate when the extraction step is omitted.

As mentioned earlier, common antibody-based techniques such as ELISA and RIA rely on an extraction step for performing precise measurements of melatonin levels. We performed a set of analyses to confirm that the developed E-aptasensor can measure melatonin concentration in untreated plasma samples without the need for an extraction step and obtain a calibration curve in plasma. First, extracted and unextracted pooled plasma (EP and UEP) samples were tested with the ELISA method. This ELISA experiment was outsourced to another laboratory, and its results are shown in **Fig. S14a**. As expected, there is a significant overestimation of the melatonin level in UEP sample compared to EP one. To investigate the effect of the extraction on the performance of the developed E-aptasensor, first the signal obtained from untreated characterized human plasma samples was corrected using a correction factor (except for the three outliers and sample JSV1225038A, which didn’t have a definite concentration). This correction factor was the ratio of the maximum current of the Hill-Langmuir (HL) binding curves (**Figs. 5b** and **5g**, also shown in **Fig. S14b**) of buffer to plasma. After correcting the plasma signals using the correction factor, the corrected signals were fed to the buffer calibration plot (inset of **Fig. 5b**), and the corresponding concentrations were extracted. **Fig. S14c** shows the absolute percentage error for the concentration of untreated plasma samples obtained from the buffer calibration plot when compared to the concentration of the same samples measured using RIA after performing an extraction step. To better show the performance of the E-aptasensor, **Fig. S14d** compares the percentage error for extracted and unextracted concentrations of pooled plasma samples measured by ELISA with the average percentage error for unextracted concentrations measured by E-aptasensor and extracted concentrations measured by RIA. As can be seen, the absolute error for the E-aptasensor is much less compared to ELISA. This exploratory comparison suggests that reasonable agreement may still be achievable even without matrix-matched calibration. However, the most appropriate and reproducible analytical approach remains the direct, plasma-matched calibration presented in the main manuscript.

**Figure S14.** Exploratory analysis comparing the direct untreated-plasma measurement, buffer calibration, and ELISA/RIA measurements. a) Melatonin concentration in extracted and unextracted human pooled plasma samples obtained by ELISA. b) HL binding curves of the aptameric assay in buffer and human plasma. c) Absolute percentage error for the concentration of untreated plasma samples obtained from the buffer calibration plot. d) Comparison of the percentage error for ELISA and the developed E-aptasensor when compared to RIA.

**Figure S15.** a) Baseline current of the biosensor over 10 days of storage at different temperatures. b) Normalized response (to saturating melatonin concentration) of the biosensors tested in the storage experiments; numbers indicate the percentage of the original response retained. c) The normalized response of the biosensor in different media at various temperatures. d) CVs of the aptameric biosensor recorded at different scan rates; the inset shows peak current as a function of scan rate. Error bars represent the normalized variability from n = 3 independent experiments.

**Figure S16.** SWV voltammograms for a set of tests performed using A2 aptamer (25 nM) on separate electrodes. Melatonin concentration was in the range of 1 pM to 3 nM.

Additive manufacturing and stencil lithography techniques can offer effective solutions to persistent issues linked with traditional microfabrication methods (e.g., photoresist coating, photolithography, solvent-based lift-off) on a large scale [19, 20]. Based on **Fig. S17a**, the stencil (a vinyl adhesive film with 0.5 mm thickness, featuring the electrode and contact patterns) was designed and then cut by a cutting plotter device. Then, it was taped onto a substrate (flexible substrates such as polystyrene and polymethyl methacrylate). Subsequently, the gold was deposited on the surface by employing the sputtering method. Finally, the stencil was removed to obtain the patterned electrodes and their contacts. Employing this method makes it possible to easily design and fabricate any configuration of electrodes in a desirable multiplexed manner. **Fig. S17b** shows a bunch of GWEs fabricated by the suggested method within about 30 minutes, meaning an efficient mass production. **Figs. S17c-e** show the characterization of the developed E-aptasensor (using homemade GWEs), including reproducibility, repeatability, and stability and its comparison with the commercial SPGEs.

**Figure S17.** Fabrication process and characterization of homemade GWEs. a) The fabless fabrication approach, b) the efficient mass production of a bunch of electrodes, c) Reproducibility of homemade GWEs and commercial SPGEs (Quantity: 20) while the aptameric assay is implemented into them, d) repeatability, and e) stability of the homemade GWEs.

**Table S3**. Sample ID and the concentration (measured by RIA) of characterized human plasma samples used in this study.

| **Sample ID** | **Concentration measured by RIA (pM)** |
| --- | --- |
| JSV1225038A | < 2 |
| JSV1187847A | 9.03 |
| JSV1142869A | 29.799 |
| JSV1175747A | 41.323 |
| JSV1187952A | 58.265 |
| JSV1225161A | 110.768 |
| JSV1225133A | 129.301 |
| JSV1142783A | 157.681 |
| JSV1187923A | 194.876 |
| JSV1259621A | 236.371 |
| JSV1142831A | 256.538 |
| JSV1194759A | 315.491 |

**References**

[1]	Z. Watkins, A. Karajic, T. Young, R. White, and J. Heikenfeld, "Week-long operation of electrochemical aptamer sensors: new insights into self-assembled monolayer degradation mechanisms and solutions for stability in serum at body temperature," *ACS sensors,* vol. 8, no. 3, pp. 1119-1131, 2023.

[2]	Y. Xiao, A. A. Lubin, A. J. Heeger, and K. W. Plaxco, "Label‐free electronic detection of thrombin in blood serum by using an aptamer‐based sensor," *Angewandte Chemie,* vol. 117, no. 34, pp. 5592-5595, 2005.

[3]	S. M. Silva, S. Hoque, V. R. Gonçales, and J. J. Gooding, "The impact of the position of the redox label on charge transfer and hybridization efficiency at DNA interfaces," *Electroanalysis,* vol. 30, no. 7, pp. 1529-1535, 2018.

[4]	S. Khaliliazar *et al.*, "Electrochemical detection of genomic DNA utilizing recombinase polymerase amplification and stem-loop probe," *ACS omega,* vol. 5, no. 21, pp. 12103-12109, 2020.

[5]	V. Mirceski, S. Komorsky-Lovric, and M. Lovric, *Square-wave voltammetry: theory and application*. Springer, 2007.

[6]	D. J. Kennaway, "A critical review of melatonin assays: Past and present," *Journal of pineal research,* vol. 67, no. 1, p. e12572, 2019.

[7]	D. J. Kennaway, "Measuring melatonin by immunoassay," *Journal of Pineal Research,* vol. 69, no. 1, p. e12657, 2020.

[8]	P. Sunon and K. Ngamchuea, "Methylene blue molecularly imprinted polymer for melatonin determination in urine and saliva samples," *Microchimica Acta,* vol. 190, no. 9, p. 348, 2023.

[9]	B. Sun, W. Sun, Z. Wang, B. Zhao, and S. Yang, "Highly sensitive electrochemical detection of melatonin based on graphene-assisted molecular imprinting technology," *Carbon Letters,* vol. 34, no. 1, pp. 437-444, 2024.

[10]	M. Pundir, L. Lobanova, S. Papagerakis, X. Chen, and P. Papagerakis, "Colorimetric sensing assay based on aptamer-gold nanoparticles for rapid detection of salivary melatonin to monitor circadian rhythm sleep disorders," *Analytica Chimica Acta,* vol. 1279, p. 341777, 2023.

[11]	K. Bahend, N. K. Bakirhan, M. El Fazdoune, A. El-Asri, E. A. Bazzaoui, and M. Bazzaoui, "Nanostructured electrochemical platform for sensitive detection of melatonin in human serum and tablets," *Synthetic Metals,* vol. 309, p. 117750, 2024.

[12]	M. Pundir, L. Lobanova, P. Papagerakis, X. Chen, and S. Papagerakis, "Competitive enzyme linked aptamer based assay for salivary melatonin detection," *Scientific Reports,* vol. 15, no. 1, p. 14276, 2025.

[13]	M. V. B. Filho, M. L. G. Agneis, M. C. de Souza, F. R. F. Seiva, and V. A. Pedrosa, "Rapid and sensitive electrochemical detection of melatonin in complex biological matrices," *Journal of Applied Electrochemistry,* vol. 55, no. 11, pp. 3011-3020, 2025.

[14]	A. Ramasubramanya, P. Singh, K.-C. Lin, S. Prasad, and S. Muthukumar, "CIRCA: Circadian inference of rhythmicity using comparative analysis from non-invasive continuous measurements of cortisol and melatonin in passive perspiration," *Biosensors and Bioelectronics: X,* vol. 26, p. 100656, 2025.

[15]	T. Ou *et al.*, "Highly uniform aptamer monolayers modified silicon nanowire field-effect transistor biosensors for the detection of melatonin as sleep disorder biomarker," *Sensors and Actuators B: Chemical,* p. 139166, 2025.

[16]	F. Shi *et al.*, "A ratiometric imprinted electrochemical sensor for highly sensitive and specific melatonin detection in biofluids," *Talanta,* p. 129023, 2025.

[17]	R. J. White, N. Phares, A. A. Lubin, Y. Xiao, and K. W. Plaxco, "Optimization of electrochemical aptamer-based sensors via optimization of probe packing density and surface chemistry," *Langmuir,* vol. 24, no. 18, pp. 10513-10518, 2008.

[18]	T. Uzawa, R. R. Cheng, R. J. White, D. E. Makarov, and K. W. Plaxco, "A mechanistic study of electron transfer from the distal termini of electrode-bound, single-stranded DNAs," *Journal of the American Chemical Society,* vol. 132, no. 45, pp. 16120-16126, 2010.

[19]	H. N. Chan, M. J. A. Tan, and H. Wu, "Point-of-care testing: applications of 3D printing," *Lab on a Chip,* vol. 17, no. 16, pp. 2713-2739, 2017.

[20]	S. G. Yedire *et al.*, "Additive manufacturing leveraged microfluidic setup for sample to answer colorimetric detection of pathogens," *Lab on a Chip,* vol. 23, no. 18, pp. 4134-4145, 2023.
